# Hyphal Fusions Enable Efficient Nutrient Distribution in *Colletotrichum graminicola* Conidiation and Symptom Development on Maize

**DOI:** 10.3390/microorganisms10061146

**Published:** 2022-06-01

**Authors:** Daniela Elisabeth Nordzieke

**Affiliations:** Genetics of Eukaryotic Microorganisms, Institute of Microbiology and Genetics, University of Göttingen, Grisebachstr. 8, 37077 Göttingen, Germany; dnordzi@gwdg.de

**Keywords:** *Colletotrichum graminicola*, oval conidia, falcate conidia, germling fusion, conidiation, autophagy

## Abstract

Hyphal and germling fusion is a common phenomenon in ascomycetous fungi. Due to the formed hyphal network, this process enables a coordinated development as well as an interaction with plant hosts and efficient nutrient distribution. Recently, our laboratory work demonstrated a positive correlation between germling fusion and the formation of penetrating hyphopodia on maize leaves outgoing from *Colletotrichum graminicola* oval conidia. To investigate the probable interconnectivity of these processes, we generated a deletion mutant in *Cgso*, in which homologs are essential for cellular fusion in other fungal species. However, hyphopodia development was not affected, indicating that both processes are not directly connected. Instead, we were able to link the cellular fusion defect in ∆*Cgso* to a decreased formation of asexual fruiting bodies of *C. graminicola* on the leaves. The monitoring of a fluorescent-labelled autophagy marker, eGFP-CgAtg8, revealed a high autophagy activity in the hyphae surrounding the acervuli. These results support the hypothesis that the efficient nutrient transport of degraded cellular material by hyphal fusions enables proper acervuli maturation and, therefore, symptom development on the leaves.

## 1. Introduction

*Colletotrichum graminicola* (Ces.) G.W. Wils., originally provided by R. L. Nicholson, Purdue University, IN, is a hemibiotrophic plant pathogen. It belongs to the globally distributed genus *Colletotrichum*, which approximately comprises 14 species complexes consisting of a total of 250 species [1,2]. As the members of this genus are able to infect virtually any plant and can cause substantial losses of fruit, vegetables and cereals, this group ranks in the top 10 of important plant pathogenic fungi [3]. *C. graminicola* belongs to the *graminicola–caudatum* complex, which specializes in the infection of a wide variety of grasses including important crops such as maize and sorghum [1,4,5]. This fungus causes corn anthracnose disease in several *Zea mays* tissues such as leaves (anthracnose leaf blight, ALB), stems (anthracnose stalk rot, ASR) and roots, and can cause systemic plant infections [6,7]. Combined with a high epidemic spreading potential, this pathogen is estimated to cause crop losses of up to 100% per field, corresponding with USD 420 million annually (USA and Canada) [8,9,10]. Two morphological distinct asexual spores, oval and falcate conidia, are responsible for *Z. mays* infections.

Falcate conidia are sickle-shaped spores formed on infected leaves by short conidiophores in asexual fruiting bodies, the acervuli [11,12]. Further typical structures in acervuli are setae, which are spike-like, highly melanized hyphae. An early study showed that setae formation correlates with humidity [13], implying a role in the moistening of acervuli and probably the spread of disease. To prevent the germination of falcate conidia directly in their formation locus, these spores secrete mycosporines, potent self-inhibitors of germination [14,15]. In contrast, oval conidia are absent from acervuli, but constricted from short hyphae during colonization and probably serve the distribution within the host plant [12,16]. Intriguingly, these conidia lack the dormant phase of falcate conidia but germinate readily under nutrient starvation and high spore densities, conditions that prevent the germination of falcate conidia [15]. In addition to germination, nutrient starvation promotes the formation of a germling network by conidial anastomosis tubes (CATs) amongst oval spores, a process so far unobserved for falcate conidia [15]. Only germlings derived from oval conidia are attracted by a gradient of glucose, also indicating differences in signal perception processes [17].

Germling fusion by the formation of CATs is a common process in ascomycetous fungi. After its first description for *Colletotrichum lindemuthianum* by Roca in 2003, this spore density-dependent process has been described for numerous fungal species [18]. Overall, it can be divided into three subprocesses: (i) the recognition of a probable fusion partner (CAT induction); (ii) the directed, alternating growth towards the fusion partner (CAT homing); and (iii) contacting with the counterpart followed by pore formation and merging (CAT fusion) [19]. All of these steps are regulated by a specific set of proteins and include mitogen-activated protein kinase (MAPK) pathways as well as Ca^2+^ and ROS signaling [18,20]. Most prominent amongst the regulating proteins are the So and MAK-2 proteins, which were first described in *Neurospora crassa* [21,22]. Both proteins are recruited in an oscillatory manner to the fungal tip during chemotropic homing, a process that is translated as alternating between ‘speaking and listening’ [18,22,23]. As research in the last several years has shown, both proteins are part of two different MAPK cascades in fungi, the pheromone response pathway (MAK-2) and the cell wall integrity pathway (CWI), in which So serves as a scaffolding protein [22,24]. Based on experiments with a *so* deletion strain in *N. crassa*, it was hypothesized that the corresponding protein is crucial for signal secretion [18,20]. As several other studies have indicated, the fusion signal might be conserved amongst fungi and can induce chemotropic interactions in distantly related species such as *Botrytis cinerea* and *N. crassa* [25]. The inter-species formation of germling fusion has also been shown to promote genetic variability in several *Colletotrichum* species [26,27].

In a recent study, we demonstrated that the ability to form germling fusions by oval conidia has severe consequences for the leaf infection process [15]. When high-spore inocula of oval conidia are applied to a maize leaf, CATs and hyphopodia formation are induced. A 10x reduction of the spore density reduces CATs as well as hyphopodia formation, indicating a probable interconnectivity of both processes. To investigate whether the fusion process affects *C. graminicola* maize infections, we generated a targeted deletion mutant in the *so* gene in this fungus. As the detailed analyses of the infected leaves showed, this germling fusion-deficient mutant also developed hyphopodia in a spore density-dependent manner. These results indicated that both processes, although relying on colony density, are not directly connected. Through the monitoring of the symptom development of ∆*Cgso* at 5 dpi, a strong reduction in acervuli formation on the leaves and on axenic culture was noted. Microscopic analyses of this conidiation defect revealed that a high number of hyphae from the *C. graminicola* wildtype and the ∆*Cgso* mutant strain showed vacuolized and empty compartments in the regions of acervuli development. In the wildtype, hyphal fusions bridged the empty compartments; in ∆*Cgso*, the developing acervuli were isolated from the living mycelium. Whilst tracking the role of the vacuolized compartments, we performed localization studies using the green fluorescent autophagy marker protein eGFP-CgAtg8. These studies showed that *C. graminicola* actively degraded its own cellular material in acervulus-forming regions. In such a setup, the developmental defect in a ∆*Cgso* strain might be caused by an abolished distribution of degraded cellular material, resulting in insufficient acervulus nutrition.

## 2. Materials and Methods

### 2.1. Strains, Media and Growth Conditions

The wildtype strain CgM2 (M1.001) of *C. graminicola* (Ces.) G.W.Wilson was used in this study [2,28]. For the generation of falcate conidia, *C. graminicola* was grown on oat meal agar (OMA) for 14–21 d at 23 °C [15]. Oval conidia as a basis for *C. graminicola* transformation and experimental procedures were obtained in liquid complete medium (1 L: 10 g glucose, 1 g yeast extract, 1 g peptone, 10 mL of solution A (500 mL: 50 g Ca(NO_3_)_2_), 10 mL of solution B (500 mL: 10 g KH_2_PO_4_, 12.5 g MgSO_4_, 2.7 g NaCl)) with 0.5 M of sucrose (CMS). After shaking the cultures for two days (80 rpm, 23 °C), the incubation was continued for 5–8 days incubation in the dark [15]. Microscopy of developing acervuli was performed from cultures grown on microscopic slides coated with a reduced OMA medium (OMA_red_). For the preparation of OMA_red_, 20 g of oat meal (Oat meal Feinblatt, Alnatura) were boiled in 500 mL of distilled water for 20 min. After cooling, the watery part of the oat meal suspension was filtrated through a cloth (Miracloth, EMD Millipore Corp., Billerica, MA, USA), filled with distilled water up to 1 L and supplemented with 15 g of agar–agar per liter. For selection of transformants, complete medium (CM) containing hygromycin B (500 µg/mL, hyg; EMD Millipore Corp., Billerica, MA, USA), nourseothricin-dihydrogen sulphate (150 µg/mL, nat; Jena Bioscience GmBH, Jena, Germany) or geneticin disulphate (G418, 400 µg/mL, gen; Carl Roth GmbH + Co. KG, Karlsruhe, Germany) were used. For cloning, *Escherichia coli* strain MACH1 (Thermo Fisher Scientific, C862003, Waltham, MA, USA) was used in standard culture conditions [29].

### 2.2. Generation of Plasmids

Plasmids were generated via the NEBuilder HiFi DNA Assembly Cloning Kit (New England Biolabs, Ipswich, MA, USA) according to the instruction manual. Information about all primer sequences, plasmids and strains are provided in Tables S1–S3.

For the generation of the *Cgso* (GLRG_01399) deletion construct, three fragments were amplified for the assembly with pJet1.2 (Thermo Fisher Scientific) using the NEBuilder HiFi DNA Assembly Cloning Kit (New England Biolabs). Approximately 1 kb of *Cgso* 5′ and 3′ regions were amplified using the primer pairs so_P_fw/so_P_rv (1049 bp) and so_T_fw/so_T_rv (1111 bp) from CgM2 genomic DNA. Amplification of *hph* cassette mediating the resistance to hygromycin B was performed using primer pair hph-f/hph-r (1417 bp) with the plasmid pRS-hyg as template [30]. By the assembly of the three fragments with pJet1.2, the plasmid pCgso_KO was generated. 

For the generation of the plasmid pCgso_c_nat used for complementation of a ∆*Cgso* null mutant, two fragments were obtained via PCR. For the amplification of *Cgso* including 5′ and 3′ regions, the primer pair so_P_comp_fw/so_T_comp_rv (59015 bp) was used. For further subcloning, *Eco*RV restriction sites were integrated in the sequences of both oligonucleotides. The *nat* cassette, mediating resistance to nourseothricin, was amplified from pRS_nat [31] using the primer pair nat-1r/PtrpC_pJet (943 bp). Both fragments were assembled with pJet1.2 using NEBuilder HiFi DNA Assembly Cloning Kit (New England Biolabs). Outgoing from pCgso_c_nat, the plasmid pJet_nat harboring the *nat* resistance cassette was generated via hydrolysis with *Eco*RV and subsequent ligation with the plasmid backbone.

To allow for the visualization of autophagy in *C. graminicola*, the plasmid peGFP-Cgatg8_gen was constructed. Three fragments were amplified using PCR. The *Cgatg8* 5′ region (Atg8_P_fw/Atg8_P_rv = 1024 bp) and the *Cgatg8* gene (GLRG_08058) including the 3′ terminator region (Atg8_wostart_fw/Atg8_T_rv = 1646 bp) were generated from CgM2 gDNA, and eGFP was amplified from plasmid p1783-1 [32] using the oligonucleotides GFP-f/GFP-r = 716 bp. 

Primers were synthesized by Sigma-Aldrich Chemie GmbH (Taufkirchen, Germany). DNA sequencing of the plasmids was performed by Microsynth Seqlab GmbH (Göttingen, Germany).

### 2.3. Transformation of C. graminicola Strains

Prior to transformation in CgM2, all plasmids were linearized (pCgso_KO: *Hind*III and *Not*I, pCgso_c_nat: *Pvu*I, peGFP-Cgatg8_gen: *Mun*I). Protoplasts were obtained from oval conidia of wildtype CgM2 (transformation of pCgso_KO and peGFP-Cgatg8) or ∆*Cgso* (transformation of pCgso_c_nat) by cell wall digestion using lysis enzyme of *Trichoderma harzianum* as described previously [33]. To obtain homokaryotic transformants, colonies that had developed on CM plates supplemented with 500 µg/mL hygromycin B (pCgso_KO), 150 µg/mL nourseothricin-dihydrogen sulphate (pCgso_c_nat), and 400 µg/mL geneticin disulphate (peGFP-Cgatg8_gen) were allowed to conidiate on OMA. After single spore isolation using the generated falcate conidia, resistant transformants were verified by PCR and Southern Blotting.

To identify *Cgso* null mutant strains, genomic DNA (gDNA) of the transformants growing on CM plates supplemented with 500 µg/mL hygromycin B was isolated and initially analyzed by PCR using the primer pair so_P_fw/so_T_rv (CgM2: 5.9 kb, ∆*Cgso*: 3.5 kb). Prior to verification of ∆*Cgso* via Southern Blot hybridization, gDNA was hydrolyzed with *Sac*I, resulting in specific bands after probe hybridization of 1303 bp (CgM2) and 6242 bp (∆*Cgso*). A *Cgso*-specific probe was obtained with the primer combination so_T_fw/so_T_rv (Figure S1).

To confirm successful ectopic integration of pCgso_c_nat and peGFP-Cgatg8_gen, PCRs were performed with the primer pairs so_seq_fw2/so_seq_rv2 and GFP-r/Atg8_P_fw, respectively. 

### 2.4. Growth and Conidiation Analyses

To examine the growth rates and conidiation of *C. graminicola* CgM2, ∆*Cgso*, and ∆*Cgso*_c, precultures were grown on CM with the appropriate antibiotics. For growth rate analyses, fresh CM plates were inoculated with a defined mycelial plug (Ø 9 mm) from the corresponding preculture plate an incubated at 23 °C. At days 3–7 after inoculation, colony size was recorded using a scanner (Epson Perfection V600 Photo, Epson, Tokyo, Japan) every 24 h. From the obtained pictures, the growth area of the single colonies derived from at least six biological replicates was determined using Fiji (mark area → measure; [34]). From these, growth rates were calculated as the difference of the growth area of two subsequent days. To examine morphological differences and conidiation of falcate conidia, CgM2, ∆*Cgso* and ∆*Cgso*_c were inoculated on OMA and CM plates outgoing from the obtained precultures with a defined mycelial plug (Ø 9 mm) and incubated at 23 °C for 21 d. Prior to harvest of falcate conidia, pictures of the overgrown plates were taken as described above. Harvest of falcate conidia was done using 0.02% Tween 20. After centrifugation for 10 min, 4000 rpm, the supernatant was discarded and the falcate conidia resuspended with 3 mL of 0.01% of Tween 20. From these solutions, numbers of spores and final volume were determined, serving as the basis for the calculation of total falcate conidia generated per plate in a total of six experiments. Conidiation of oval conidia was determined in flasks containing liquid 100 mL CMS cultures inoculated with five mycelial plugs each (Ø 9 mm) derived from the CM precultures. After shaking for 2 d at 80 rpm, cultures were further incubated for 6 d in darkness (23 °C). To separate oval conidia from mycelia, the cultures were filtered through a sterile cloth (Miracloth, EMD Millipore Corp., Billerica, MA, USA). The flow through was centrifuged for 10 min, 4000 rpm and the pellet was resuspended in 500 µL of distilled water. From these solutions, the numbers of spores and the final volume were determined, serving as the basis for the calculation of total oval conidia generated per flask in a total of six independent experiments.

### 2.5. Quantification of Conidial Anastomosis Tube (CAT) Formation

For the examination of germling fusion formation, 50 µL of oval conidia having the concentration of c = 5 × 10^7^ mL^−1^ were spread on nutrient poor water agar (1% Serva Agar, 1% agarose, 25 mM NaNO_3_) and incubated at 23 °C for 17 h as described previously [15]. For each of the three independent replicates, at least 100 conidia were examined for fusion with other conidia or hyphae. 

### 2.6. Acervuli Development

For the analysis of acervuli development, CgM2, ∆*Cgso*, ∆*Cgso*_c, and CgM2::eGFP-Cgatg8 precultures were grown on CM with the appropriate antibiotics. From these, mycelial plugs were transferred to microscopic slides overlaid with OMA_red_ and incubated for 5 d at 23 °C. From the strains CgM2, ∆*Cgso*, ∆*Cgso*_c pictures were taken from hyphae, which showed the starting of acervulus development. Since in progressed acervuli a massive number of conidiophores cover the leading hyphae and hamper microscopic evaluations of those, regions with single setae and a minor number of conidiophores were selected for analysis. Depending on the orientation of the hypha, a length of 266.19–356.29 µm was evaluated for the presence of hyphal fusions, empty or vacuolized hyphal compartments for a total of 30 hyphae of all strains. Different layers of developing avervuli were recorded in a fixed distance of 1 µm and processed with Fiji (Image → Stacks → Images to Stacks; Image → Stacks → Z-Projection (sum slices); [34]).

### 2.7. Microscopy

Microscopic documentation was performed with the AxioImager M1 microscope (Zeiss, Jena, Germany) with differential interference contrast (DIC). Image capturing was performed with a Photometrix CoolSNAP HQ camera (Roper Scientific, Photometrics, Tucson, AZ, USA). Images were processed using ZEISS ZEN Digital Imaging (version 2.3; Zeiss). For visualization of expressed green-fluorescent *CgAtg8*, Chroma filter set 49002 (exciter ET470/40x, ET525/50m, beamsplitter T495lpxr) was used. For each experiment, at least three biological replicates were analyzed.

### 2.8. Plant Infection-Related Assays

The *Zea mays* cultivar Mikado (KWS SAAT SE, Einbeck, Germany) was grown as described previously at a day-night cycle (12 h light:12 h dark, 26 °C:18 °C) in a PK 520 WLED plant chamber (Poly Klima Climatic Growth System, Freising, Germany) [15]. To determine the formation of infection structures and symptom development after 1 or 5 dpi, secondary leaves from 16 days old maize plants were cut from the plant and fixed on top of a wet blotting paper (BF2 580 × 600 mm, Sartorius, Göttingen, Germany). 10 µL drops of conidia suspension in 0.01% Tween 20 solutions in final inocula of 10^3^ and 10^2^ were applied onto leaves and incubated at 22 °C. To examine the early events of plant infection, the experiment was stopped 24 h after inoculation and the leaves bleached in 100% ethanol as described previously [15]. The rates of infection structures and germling fusion per conidium were examined for at least three different infection areas. Different layers of infected leaves were recorded in a fixed distance of 1 µm and processed with Fiji (Image → Stacks → Images to Stacks; Image → Stacks → Z-Projection (sum slices); [34]). Symptom development of single inoculation spots was rated at 5 dpi using an index ranking from 1 (no symptoms), 2 (minor symptoms), 3 (symptoms) to 4 (severe symptoms) [15] for at least 40 individual spots. As negative controls, mock infections were inoculated with 10 µL of 0.01% Tween 20. To test for the penetration ability of *C. graminicola* strains, a defined inoculum (Ø 9 mm) from a preculture on CM was transferred to a single cellophane sheet (Cellophane Sheets II, 140 × 133 mm, SERVA Electrophoresis GmbH, Heidelberg, Germany) topping OMA. After incubation for 3 d at 23 °C, the cellophane was removed and further incubated for 4 d, followed by examination for colony outgrowth. All experiments were performed at least in three independent replicates. 

### 2.9. Statistics

For all experiments presented in this study, the *t*-test for unequal variances, also referred to as Welch-test [35], was used for all experiments displayed.

## 3. Results

### 3.1. A Defective Germling Fusion Process Does Not Affect Hyphopodia Formation by Oval Conidia

**Figure 1:**
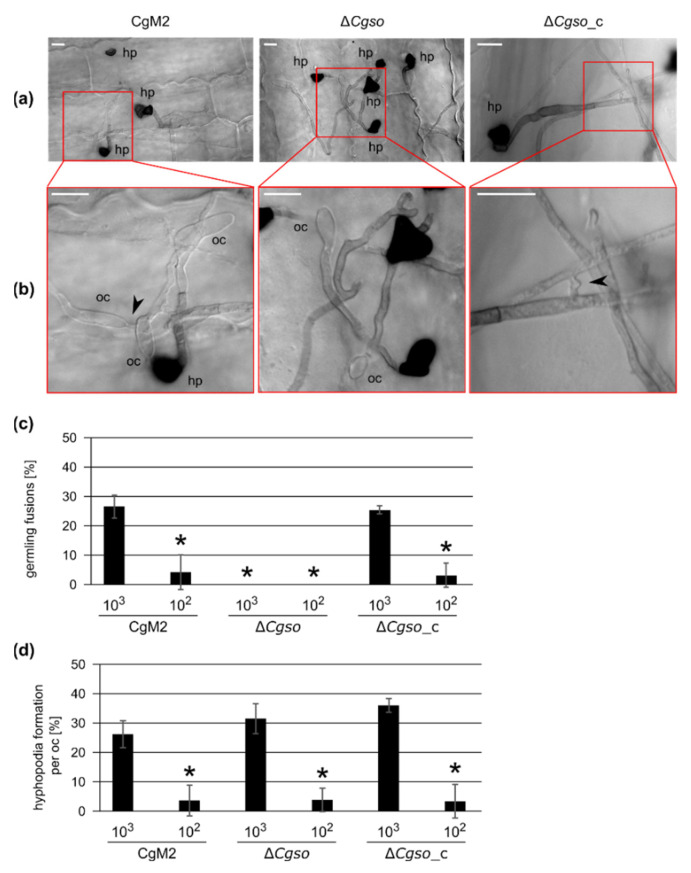
Hyphopodia and cellular fusion formation by oval conidia-derived germlings. Inoculation of secondary leaves obtained from 16 d old *Z. mays* (cv Micado) plants with 10^3^ and 10^2^ oval conidia of the depicted strains. In addition, 1 dpi, the infection process was stopped. Leaves were parted in four and de-colorized in 100% EtOH for 3 d. (**a**) overview of typical representation of colony and infection structure development with an inoculum of 10^3^ at 1 dpi, hp = hyphopodia, scale bar = 10 μm; (**b**) enlarged depiction of the indicated area of (**a**), indicating presence or absence of hyphal fusions (black arrow heads), oc = oval conidia, hp = hyphopodia, scale bar = 10 μm; (**c**,**d**) quantification of cellular fusions (**c**) or hyphopodia (**d**) formed by germlings derived from oval conidia in one inoculation spot. Error bars represent SD calculated from ≥3 experiments, * *p* < 0.05.

To investigate whether the formation of germling networks by fusions was a prerequisite for hyphopodia development by oval conidia, we generated a null mutant of *Cgso* (GLRG_01399) by replacing the native gene with an *hph* cassette, mediating the resistance to hygromycin B (Figure S1). Similar to the findings in other fungi [18], ∆*Cgso* oval conidia were not able to form CAT fusions on axenic cultures, but this process was restored by the re-integration of the *Cgso* gene (Figure S2). To examine the impact of CAT fusion deficiency on hyphopodia formation, leaf infection experiments were performed, which were aborted at 1 dpi. We inoculated several droplets per leaf containing high (10^3^) and low (10^2^) inocula of oval conidia, resulting in different spore densities on the inoculation spots. After the bleaching of the corresponding leaves, microscopic observations of the first steps of the leaf infection process were possible (Figure 1). The oval conidia of both the CgM2 wildtype and ∆*Cgso*_c complementation strain showed frequent germling fusions and hyphopodia formations in the spots inoculated with high spore densities comparable to our observations on axenic cultures (Figure S2). On the leaves inoculated with ∆*Cgso* oval conidia, no germling fusion events were observed. The formation rate of hyphopodia of ∆*Cgso*, however, was comparable with the wildtype and complementation strains in the experiments with high and low spore inocula (Figure 1).

### 3.2. ∆Cgso Shows Reduced Symptom Development on Z. mays Leaves

**Figure 2:**
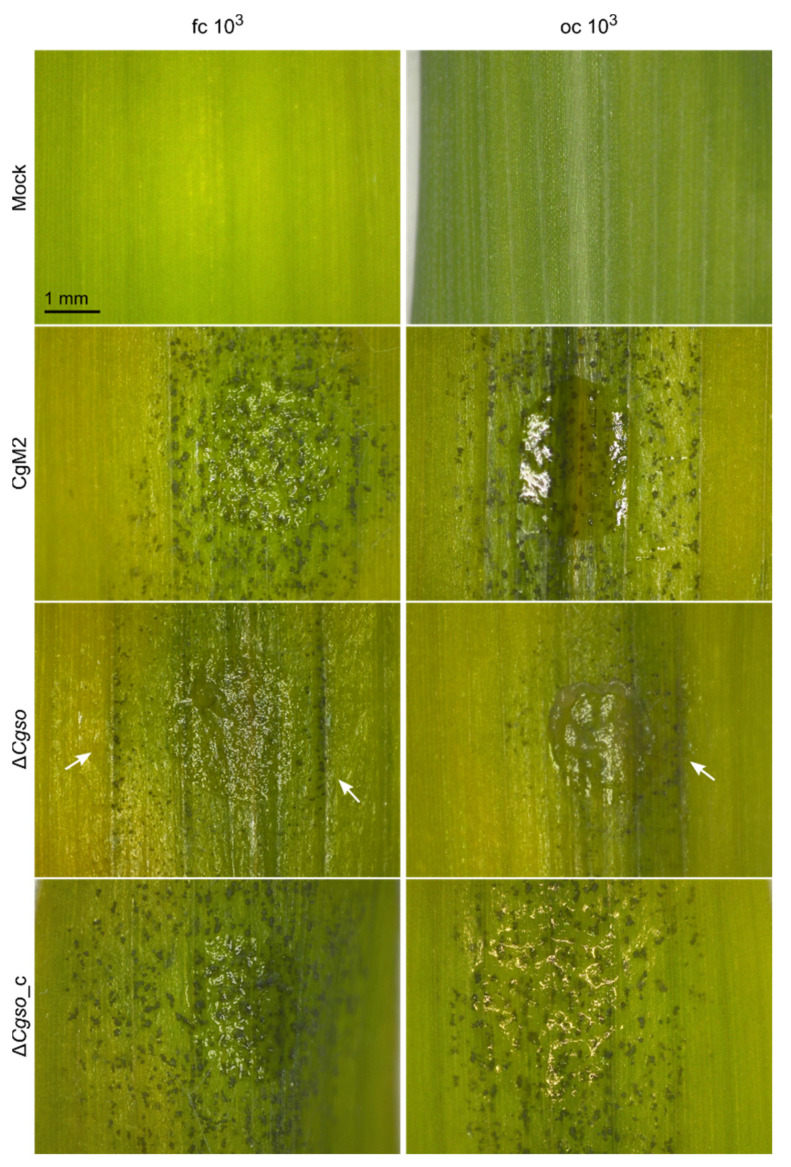
*Z. mays* leaf infection. Second leaves of 16 d old *Z. mays* plants (cv Mikado) were inoculated with droplets *C. graminicola* conidia containing 10^3^ conidia. Typical appearance of symptoms on intact leaves is depicted after incubation with falcate (fc) or oval (oc) conidia for 5 d. Arrows indicate development of acervuli along vascular bundles, scale bar = 1 mm.

To test whether ∆*Cgso* was affected in the symptom development on the leaves, infection analyses were performed with high and low spore densities of oval and falcate conidia, respectively, and evaluated at 5 dpi (Figure S3). As depicted in Figure 2, a CgM2 inoculation resulted in the development of evenly distributed acervuli at 5 dpi, which was strongest on the inoculation spot itself and spread to the surrounding areas. Chlorosis was also visible outside the inoculation spots. In contrast, the plant tissue remained green at the sides of the acervuli formation, indicating the successful penetration and manipulation of the plant metabolism by the fungus, a phenomenon also referred to as ‘green island formation’ [36]. Chlorosis and green islands were visible in ∆*Cgso*, also indicating the successful colonization of the plant by the mutant strain. However, the inoculation spots remained mostly empty of developing acervuli, indicating a conidiation defect in the *Cgso* deletion strain. The rare acervuli in ∆*Cgso* formed along the vascular bundles outside the inoculation spot (Figure S4). Interestingly, this effect was independent of the spore inoculum and was observed in the oval as well as falcate conidia infections.

A detailed comparison of the symptom developments was performed using a standardized rating system of individual inoculation spots [15]. This system equated the successful plant colonialization and formation of a new generation of falcate conidia for disease spreading with the symptom development on the leaves. For falcate conidia infections, predominantly severe symptoms developed in CgM2 as well as in ∆*Cgso*_c for both high and low spore inocula, which were indicated by a high abundance of acervuli, the formation of chlorosis and green islands. In contrast, only minor symptoms were detected in response to inoculation with ∆*Cgso* because only a few acervuli formed. In the leaf infection assays with oval conidia, we verified the previous results of our laboratory work, showing a significantly reduced symptom development when low spore densities were present (Figure 2). In the ∆*Cgso* infection experiments, this interdependence was reversed, and minor symptoms dominated both spore concentrations tested.

Comparing these results with our previous observations of hyphopodia development from oval conidia on leaves, the differences in the hyphopodia formation rate in CgM2 and ∆*Cgso*_c were well-reflected by spore inocula-dependent symptom developments (Figure 1 and Figure 2). However, the symptom developments of ∆*Cgso* did not follow this line, indicating a severe defect in the development of the mutant *in planta*. To rule out a probable penetration defect of ∆*Cgso*, a qualitative penetration assay was performed. As already indicated by the formation of green islands, no differences between the three strains tested were observed in the total of nine biological replicates (Table S4). Together, these results indicated that ∆*Cgso* is virulent and successfully able to penetrate and colonize the host plant, but is impaired in acervulus formation and conidiation, which might reduce the pathogens disease spreading capacity.

### 3.3. A Cgso Deletion Mutant Is Drastically Reduced in the Conidiation of Falcate Spores

**Figure 3:**
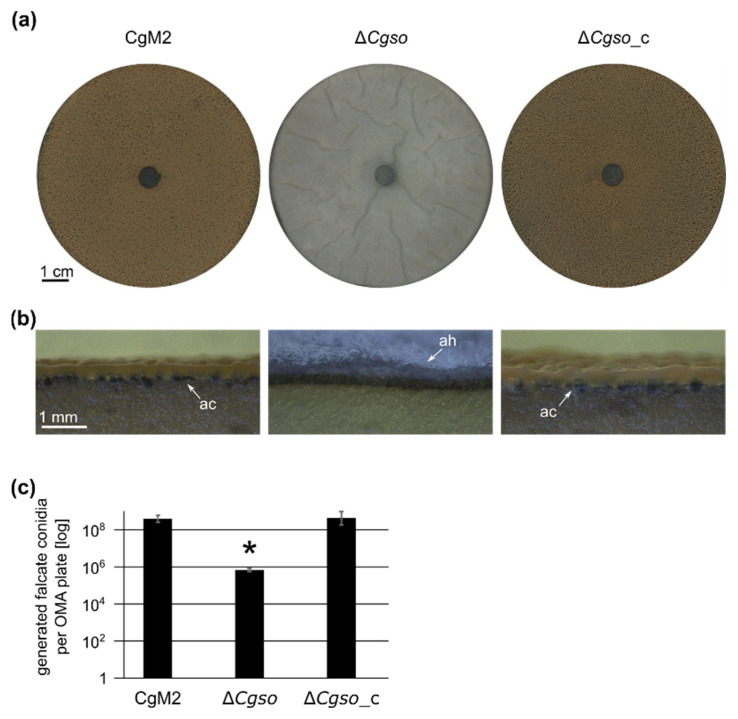
Generation of falcate conidia in *C. graminicola* strains. *C. graminicola* CgM2 (wildtype), Δ*Cgso* deletion strain as well as Δ*Cgso* with a re-integrated *Cgso* gene including native 5’ and 3’ regions (Δ*Cgso*_c) were incubated for 21 d on oatmeal agar (OMA) plates at 23 °C. (**a**) plate overview, scale bar = 1 cm; (**b**) cross section, scale bar = 1 mm, ac = acervuli, ah = aerial hyphae; (**c**) quantification of falcate conidia per plate. Values are depicted in a logarithmic scale, error bars represent SD calculated from 6 experiments, * *p* < 0.05.

To further investigate the conidiation defect of ∆*Cgso*, we grew wildtype CgM2, *Cgso* deletion and complementing strains under conditions promoting the maturation of acervuli. In contrast with the wildtype strain, which showed significant falcate conidia generation, the ∆*Cgso* deletion mutant showed the formation of arial mycelia, embedding more than a 100× reduced number of falcate conidia. This phenotype was reversed in the ∆*Cgso* strain with a re-integrated *Cgso* gene (Figure 3). To test whether the reduced conidiation of ∆*Cgso* might be caused by a vegetative growth defect, the growth rates of all three strains were compared. However, no substantial differences were observed (Figure S5). We further analyzed whether the *Cgso* deletion affected the conidiation processes of *C. graminicola* in general and compared oval conidia generation in the wildtype with the deletion strains. We found no significant differences, indicating a spore type-specific conidiation defect (Figure S6).

### 3.4. Autophagy-Degraded Cellular Material Might Serve for the Nutrition of Acervuli

**Figure 4:**
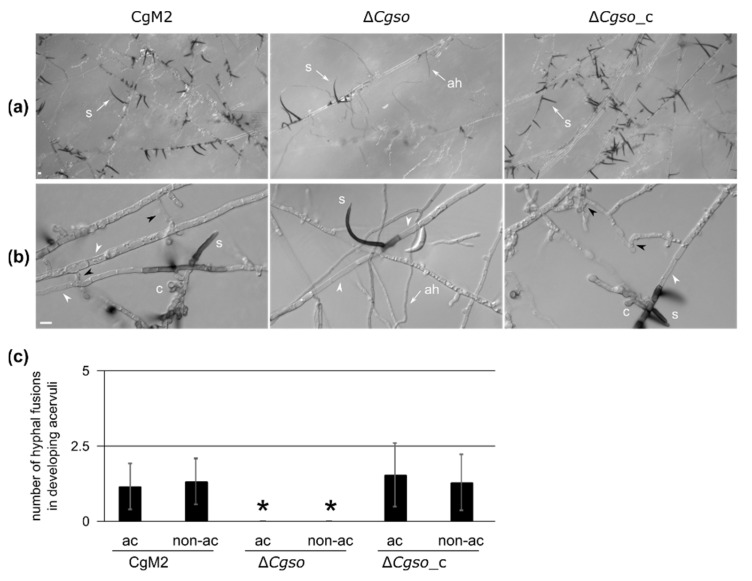
Development of young acervuli in a *Cgso* deletion strain. *C. graminicola* CgM2 (wildtype), Δ*Cgso* deletion strain as well as the corresponding complementation strain Δ*Cgso*_c were analyzed for the development of asexual fruiting bodies, the acervuli. Depicted strains were inoculated on microscopic slides covered with reduced oat meal agar (OMA_red_) for 5 d, 23 °C, ah = aerial hyphae, s = setae, hyphal fusions are indicated with black arrow heads, empty hyphal compartments with white arrow heads, scale bar = 10 μm. (**a**) overview of acervuli forming regions; (**b**) Z-projections of stack images of hyphae with developing setae and conidiophores (levels of 1 μm); (**c**) total numbers of hyphal fusions on hyphae, which show (ac) or do not show (non-ac) developing acervuli. Error bars represent SD calculated from 30 experiments, * *p* < 0.05.

As we observed a reduced number of acervuli in the cultivation media as well as on infected leaves formed by ∆*Cgso* (Figure 2 and Figure 3), we examined this process after 5 d of inoculation in a reduced OMA medium (OMA_red_). As depicted in Figure 4A, the overall density of setae, typical markers of developing acervuli, was highly reduced in ∆*Cgso*. We observed a high number of aerial hyphae in ∆*Cgso*, which are formed, as with setae, from dark-pigmented hyphal fragments. To analyze the acervuli development in detail, we examined young developing acervuli, indicated by the presence of a single setae and a reduced number of conidiophores (Figure 4B). In all strains, we observed several empty or highly vacuolized hyphal compartments separated by setae. In ∆*Cgso*, these parts appeared to separate the young acervuli from the living fungal tissue. In CgM2 and ∆*Cgso*_c, however, we monitored the frequent fusion of neighboring hyphae, again connecting the asexual fruiting bodies with the living fungal cells. To examine whether or not these processes were limited to hyphal regions with developing acervuli, we quantified the number of empty and vacuolized hyphal compartments in addition to the hyphal fusion events in the acervuli and non-acervuli regions (Figure 4 and Figure S7). No differences were observed, indicating that these were general processes occurring in a *C. graminicola* colony of that age.

**Figure 5:**
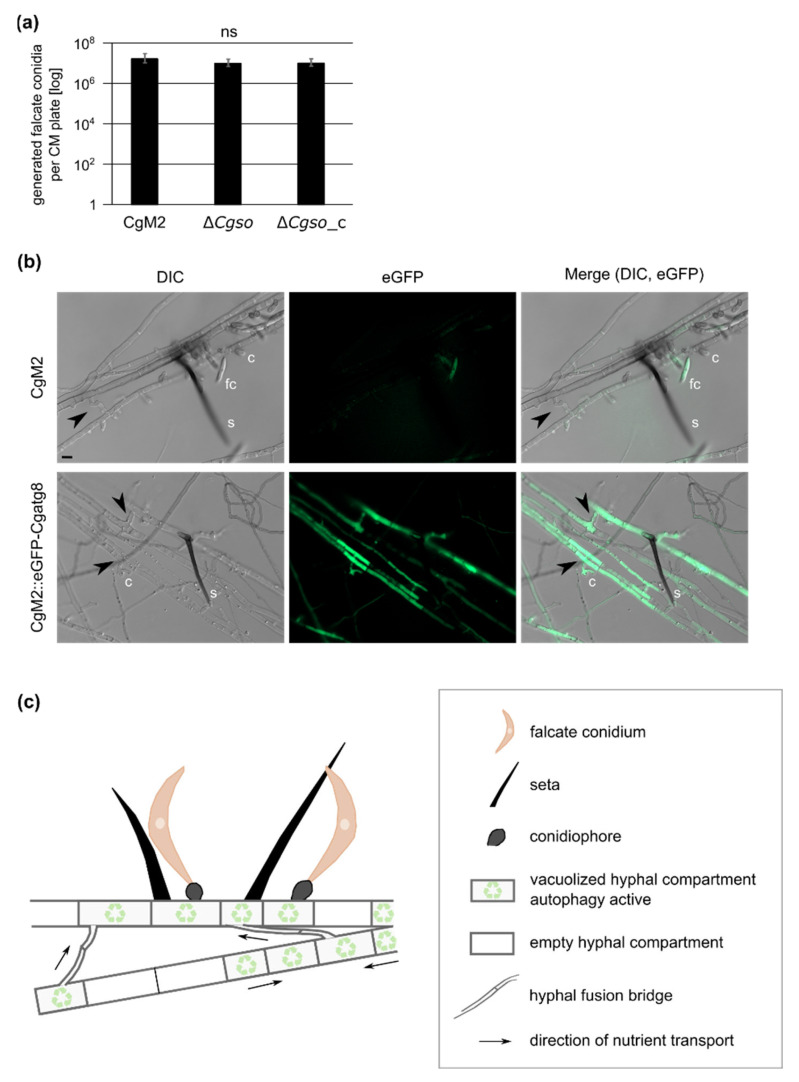
Autophagy in developing acervuli and model. (**a**) quantification of falcate conidia after growth on complex medium (CM) for 21 d, 23 °C. Values are depicted in a logarithmic scale, error bars represent SD calculated from six experiments, ns, *p* > 0.05; (**b**) *C. graminicola* wildtype strain CgM2 and CgM2::eGFP-Cgatg8 expressing green fluorescent autophagy marker CgAtg8 were inoculated on microscopic slides covered with reduced oat meal agar (OMA_red_) for 5 d, 23 °C. Selected layers from acervuli recordings with a fixed distance of 1 μm are depicted for each strain. In CgM2, falcate conidia appear green due to autofluorescence, s = setae, c = conidiophores, fc = falcate conidia, hyphal fusions are indicated with black arrow heads, scale bar = 10 μm; (**c**) optimized distribution of autophagy-recycled cellular components by hyphal fusion bridges allows for proper acervulus maturation and falcate conidia production in *C. graminicola*.

Bearing in mind the results of our leaf infection experiment in which the acervuli of ∆*Cgso* only formed along the vascular bundles, we speculated about a probable interconnection between coordinated cellular degradation and the nutrition of the developing acervuli. In such a setup, the formed hyphal fusion bridges of CgM2 and ∆*Cgso*_c might be crucial to transport the degraded cellular material to the nutrient sink. In strains defective of cellular fusion, the nutrition of acervuli would cease after an early timepoint, resulting in a reduced number of falcate conidia. To support this hypothesis, we grew all three strains in a complex medium that promoted vegetative growth but no acervuli development. The quantification of falcate conidia from these plates showed no significant differences between all three strains, suggesting an early abortion of acervuli development of the ∆*Cgso* mutant *in planta* due to nutrient limitations (Figure 5 and Figure S8).

Due to the prominent empty or vacuolized hyphal compartments of the developing acervuli, we speculated about a controlled degradation process at these sites. Autophagy is an intracellular vacuolar degradation process in eukaryotes that regulates starvation adaptation as well as developmental processes [37]. To test whether autophagy took place at the sites of developing acervuli, a strain expressing green fluorescent CgAtg8 was generated. As Atg8 proteins in other fungi can be localized in small autophagosomes as well as in larger vacuoles (depending on the age of the fungal hyphae [38]), we first checked the overall localization patterns in CgM2::eGFP-Cgatg8. As depicted in Figure S9*,* the expression of *eGFP-Cgatg8* resulted in green fluorescent autophagosomes in the young fungal filaments, whereas, in the older hyphae, we monitored a strong accumulation of the signal in the vacuoles. When monitoring the same strain after 5 d of growth on OMA_red_, we observed a bright fluorescence in the hyphae both forming and surrounding the acervulus. The fluorescent signal filled large parts of the hyphal segments, which we declared to be strongly vacuolized in our quantification analyses (Figure 4 and Figure 5B), indicating that the autophagy degradation process was highly accelerated compared with normal-aging hyphae (Figure S9). Interestingly, the formed hyphal fusion bridges also showed a strong green fluorescent signal, supporting the hypothesis that these are required for the successful transport of degraded cellular material for acervulus maturation.

## 4. Discussion

### 4.1. An Unknown Quorum-Sensing Mechanism Regulates Hyphopodia Formation from Oval Conidia 

As our investigations showed, different developmental processes were induced in *C. graminicola*, depending on the number of spores applied, showing typical characteristics of quorum-sensing (QS) processes. QS was first discovered in the 1960s in Gram-positive bacteria [39,40]. In this process, small signaling molecules (quorum-sensing molecules, QSM) are secreted, which are able to shape the behavior of the sensing microorganisms [41]. Since then, QS has been discovered for multiple bacteria as well as fungal species and is now accepted as the central mechanism of inter-kingdom communication [42,43]. In *C. graminicola*, two QS-dependent processes have been described. Falcate conidia secrete mycosporines as germination inhibitors when present in high spore numbers, as in acervuli [14,15]. The formation of germling fusions is also dependent on spore densities; this has been documented for *C. graminicola* as well as several other fungi [15,26,44,45,46]. In an earlier study, we observed a positive correlation between the formation of penetrating hyphopodia by oval conidia and the germling fusion process on leaves, indicating that the pathogenicity program of oval conidia is also dependent on the spore concentration [15]. In this study, we generated and analyzed a deletion mutant in the *C. graminicola so* gene, an essential gene for cellular fusion in other fungi. As discovered from detailed leaf microscopy, hyphopodia formation was dependent on the conidia density and independent of the ability of the investigated strain to perform a fusion or not (Figure 1). These results indicated that an unknown QS process regulated the formation of penetration structures from oval conidia. Such an involvement of QS in host infections has been shown for many pathogenic bacteria. In those processes, QS enabled the synchronized expression of the virulence factors, enabling the microbe to overcome the defense mechanisms of the host [47]. Also for the fungus *Colletotrichum coccodes*, a positive correlation between the penetration structure formation and the inoculum size has been shown [48]. One possible explanation for this phenomenon is the spore density-dependent secretion of germination enhancers, which could indirectly affect hyphopodia formation. In a recent study, the positive and negative germination regulation of the tomato wilt fungus *Fusarium oxysporum* f. sp. *lycopersici* by pheromone signaling was shown. In that fungus, the α-pheromone interaction with the Ste2 receptor led to the repression of conidial germination, whereas the interaction of the a-pheromone with the Ste3 receptor relieved repression in a cell density-dependent manner [49]. We observed no density-dependent differences in the germination patterns in *C. graminicola* oval conidia [15], indicating that the determining QS mechanism might be specific for hyphopodia development.

### 4.2. Coordinated Nutrient Recycling and Distribution Might Be the Basis for Acervulus Maturation

The proper development of distinct morphological structures in fungi such as appressoria, sexual fruiting bodies and the formation of conidiophores requires the mobilization and translocation of nutrients [50]. Seeking explanations for the drastically reduced falcate conidia production and decreased amounts of acervuli formed in the infected leaves of the ∆*Cgso* mutant strain, we monitored the developing conidiation sites in detail (Figure 4). We observed a high number of empty or heavily vacuolized compartments, which were bridged by hyphal fusions in the CgM2 wildtype strain and the ∆*Cgso*-complementing strain. From these observations, we deduced that it was likely that autophagy, a controlled degradation process in eukaryotic organisms, took place in the developing acervuli. 

In general, it is possible to distinguish between three autophagy types: microautophagy; macroautophagy; and chaperon-mediated autophagy. Fungal macroautophagy describes the formation of a double-membrane vesicle, the autophagosome, outgoing from the phagophore assembly site (PAS) [51,52,53]. Within the autophagosome, a portion of the cytoplasm containing excessive or defective proteins and organelles is engulfed [52,54]. These autophagosomes fuse with the vacuoles, resulting in the degradation of the inner autophagosomal membrane as well as its cargo by hydrolases [55,56]. Autophagy-related (Atg) proteins are responsible for the correct assembly of the PAS as well as the binding of cargo, autophagosome fusion with vacuoles and finally its degradation. In yeast, 36 Atg proteins have been described, many of which are conserved in all eukaryotes including filamentous fungi [57,58]. One of these is Atg8, a ubiquitin-like protein, of which a conjugate with lipid phosphatidylethanolamine (PE) is formed to anchor the protein to the forming autophagosomal membrane at the PAS. Therefore, Atg8 is a structural component of the autophagosome and remains bound to its membrane until its degradation in the vacuole takes place [59,60].

Recently, autophagy has been investigated in several fungal species. Phenotypic investigations of *atg* deletion mutants revealed defects in proper sexual and asexual development, vegetative growth, resistance to various stresses (nitrogen, carbon, metal ion starvation, reactive oxygen species and osmotic stress) and pathogenicity [50,57,61,62]. Several of these processes have been linked to the improper nutrition of developing structures such as appressoria, sexual fruiting bodies and conidiophores due to defects in the overall autophagy process [50]. To investigate a probable role of autophagy in acervulus development, we fused green fluorescent eGFP N-terminally to CgAtg8 and expressed the fusion protein in the wildtype CgM2. The hyphae of the resulting strain CgM2::eGFP-Cgatg8 showed typical Atg8 localization patterns known from other fungi; for example, dot-like autophagosomes in young hyphae and bright green vacuoles in older tissues (Figure S9) [38,63]. At sites of acervuli development, we monitored that the complete hyphal segments shone bright green, indicating a massive turnover due to active autophagy in these regions (Figure 5). A similar pattern was shown for fluorescent-tagged Atg8 in *Magnaporthe oryzae*. In this fungus, Atg8 was brightly visible in developing conidiophores, conidia and aerial hyphae [63]. A *Moatg8* deletion strain was heavily affected in conidiation as well as vegetative growth and virulence [64,65]. Similar defects in conidiation have also been observed for homologous *atg8* deletion mutants in various other fungi such as *Aspergillus* ssp., *Colletotrichum orbiculare*, *Ustilaginoidea virens* and *F. graminearum*, indicating a general requirement for cellular degradation for the nutrition of conidiophores [66,67,68,69,70,71]. In *U.* *virens*, the observed reduced virulence of ΔUvatg8 was explained with its decreased conidiation rate [71], similar to our observations for Δ*Cgso*.

In the same experiments using eGFP-CgAtg8 as an autophagy marker, it was obvious that, within the autophagy active regions, hyphal fusion bridges readily formed in the wildtype and ∆*Cgso*::*Cgso* strains, supporting the hypothesis that a hyphal fusion might enable the efficient distribution of the degraded cellular material to the point of need (Figure 5). Hyphal and germling fusion has been intensively studied since its first description in 2003 in *C. lindemuthianum* by Roca and coworkers [72]. Since then, several additional defects have been observed for gene deletion mutants affected in the fusion processes. For example, in the coprophilous fungus *Sordaria macrospora*, defects in the hyphal fusion process often went hand-in-hand with an aborted sexual development [24,73,74,75,76,77,78,79,80,81,82]. From this co-occurrence of phenotypes, it was deduced that the fusion process was either a prerequisite for proper sexual fusion within the maturing fruiting bodies or that the nutrition of this sexual structure was decreased. There are a few exceptions to this rule; deletion mutants in autophagy genes such as *atg8* and *atg4* are sterile, but their ability to form fusion bridges remains intact [38,83]. It is tempting to assume that fusion formation and autophagy, despite being independent, are both dedicated to enable the same development process. The results of this study support such a perspective; although ∆*Cgso* was unable to form hyphal fusions, the number of empty or vacuolized hyphal compartments in the developing acervuli did not differ from the fusion-competent CgM2 and ∆*Cgso*::*Cgso* strains (Figure S7). This perspective might also explain the reduced symptom development on the leaves (Figure 2). The wildtype CgM2 and the ∆*Cgso*-complementing strain produced acervuli predominantly at the sides with a high hyphal density *in planta* within the area of the inoculation spot. In contrast, acervuli were absent from these parts in the *Cgso*-null mutant, but formed along the vascular bundles outside the inoculation site. There, nutrient-rich phloem could further support the acervuli development and compensate for the defective nutrient distribution caused by the absence of hyphal fusions.

## 5. Conclusions

The formation of fungal networks by the fusion of hyphae and/or conidia is a typical feature of filamentous ascomycetes. In this study, we provided evidence that the formation of penetrating hyphopodia by *C. graminicola* oval conidia was independent of germling fusion formation but regulated by an unknown quorum-sensing molecule. By a combination of microscopic analyses with *Z. may* leaf infection assays, we demonstrated that hyphal fusion, probably together with autophagy, might be crucial for the efficient nutrition of asexual fruiting bodies. Thus, defects in the fusion process resulted in a decreased conidiation of falcate conidia and reduced symptom developments on the leaves, which could affect the spreading of disease from the anthracnose fungus. 

## Data Availability

The data presented in this study are available on request from the corresponding author.

## Supplementary Materials

The following supporting information can be downloaded at: https://www.mdpi.com/article/10.3390/microorganisms10061146/s1, Figure S1: Generation and verification of a *Cgso* deletion strain in *C. graminicola*; Figure S2: Germling fusion rate of *C. graminicola* strains on water agar; Figure S3: *Zea mays* leaf infection, overview; Figure S4: Details and quantification of *Z. mays* leaf infection; Figure S5: Growth rates of *C. graminicola* strains; Figure S6: Generation of oval conidia *C. graminicola* wildtype and Δ*Cgso* deletion mutant; Figure S7: Quantification of empty and vacuolized hyphal compartments in *C. graminicola* hyphae; Figure S8: *C. graminicola* falcate conidiation on complex medium; Figure S9: Localization of the autophagy marker protein CgAtg8; Table S1: Oligonucleotides used in this study; Table S2: Plasmids used in this study; Table S3: *Colletotrichum graminicola* strains used in this study; Table S4: Cellophane penetration ability of *C. graminicola* strains.
