# Supplementary material for: Hyphal Fusions Enable Efficient Nutrient Distribution in Colletotrichum graminicola Conidiation and Symptom Development on Maize
Source: Microorganisms. 2022 Jun 1;10(6):1146. doi: 10.3390/microorganisms10061146 (PMC9231406; doi:10.3390/microorganisms10061146)
Supplement: Supplementary file 1 [file microorganisms-10-01146-s001.zip › Supplementary_Material.pdf]

Hyphal Fusions Enable Efficient Nutrient Distribution in *Colletotrichum graminicola* Conidiation and Symptom Development on Maize

Figures

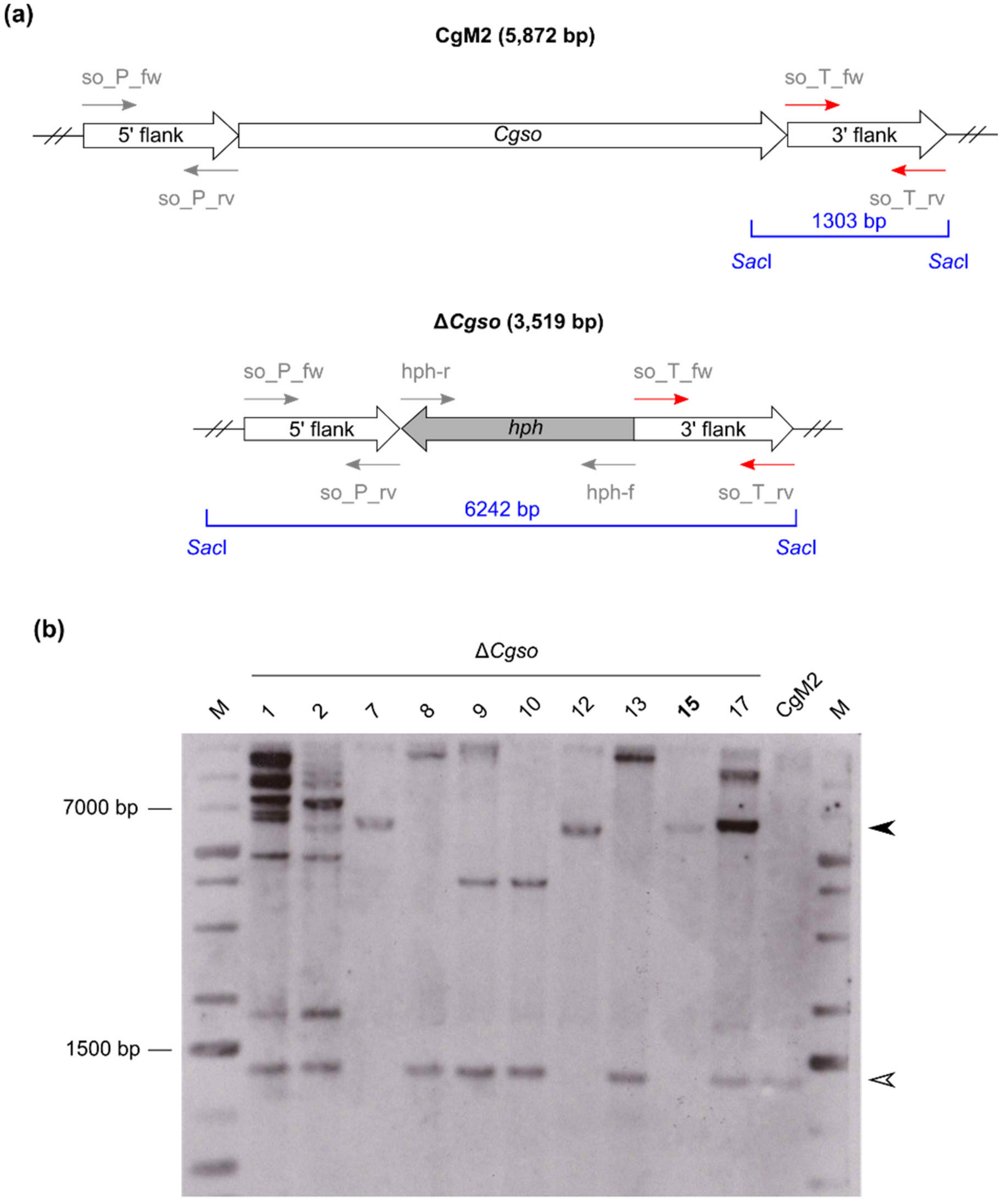

Figure S1. Generation and verification of a *Cgso* deletion strain in *C. graminicola*. (**a**) Strategy for the generation of a Δ*Cgso* mutant. Linear maps of the *Cgso* locus in the CgM2 wildtype strain and the deletion mutant. PCR binding sites for the generation of the construct and Southern Blot probe are indicated in grey and red, respectively,

recognition sites for *Sac*I are indicated in blue; (**b**) Southern Blot analysis to identify Δ*Cgso* deletion mutants. Genomic DNA of CgM2 wildtype and several Δ*Cgso* strains were hydrolyzed with *Sac*I. The verified mutant, which was further used for phenotypic analysis and complementation, is indicated in bold letters. Hybridizing bands showing the expected size for wildtype and Δ*Cgso* are indicated in white and black arrow heads, respectively, M = GeneRuler™ 1 kb Plus ladder.

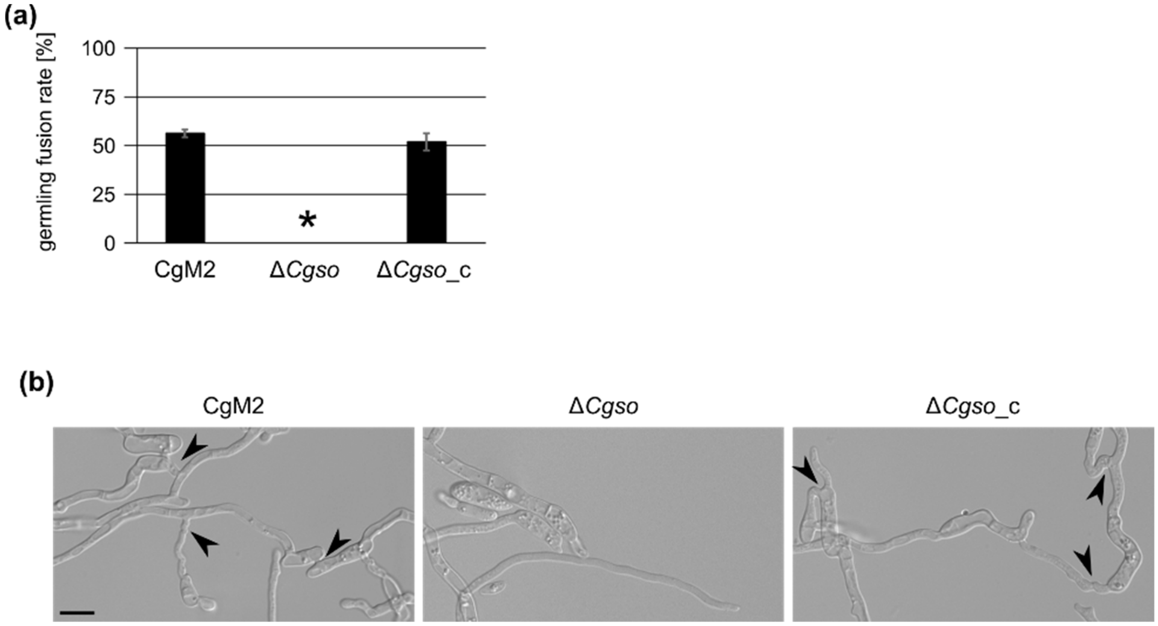

Figure S2. Germling fusion rate of *C. graminicola* strains on water agar. 50 µl of c = $5*10^7$/ml oval conidia of the depicted *C. graminicola* strains were spread on water agar (1% Serva Agar, 1% Agarose, 25 mM $NaNO_3$) and inoculated for 17 h at 23°C. (**a**) For quantification of germling fusion, a minimum of 100 oval conidia was assessed per biological replicate. Error bars represent SD calculated from 3 experiments, *, $p < 0.05$; (**b**) Representative pictures of germlings, germling fusion sites are indicated (black arrow heads), scale bar = 10 µm.

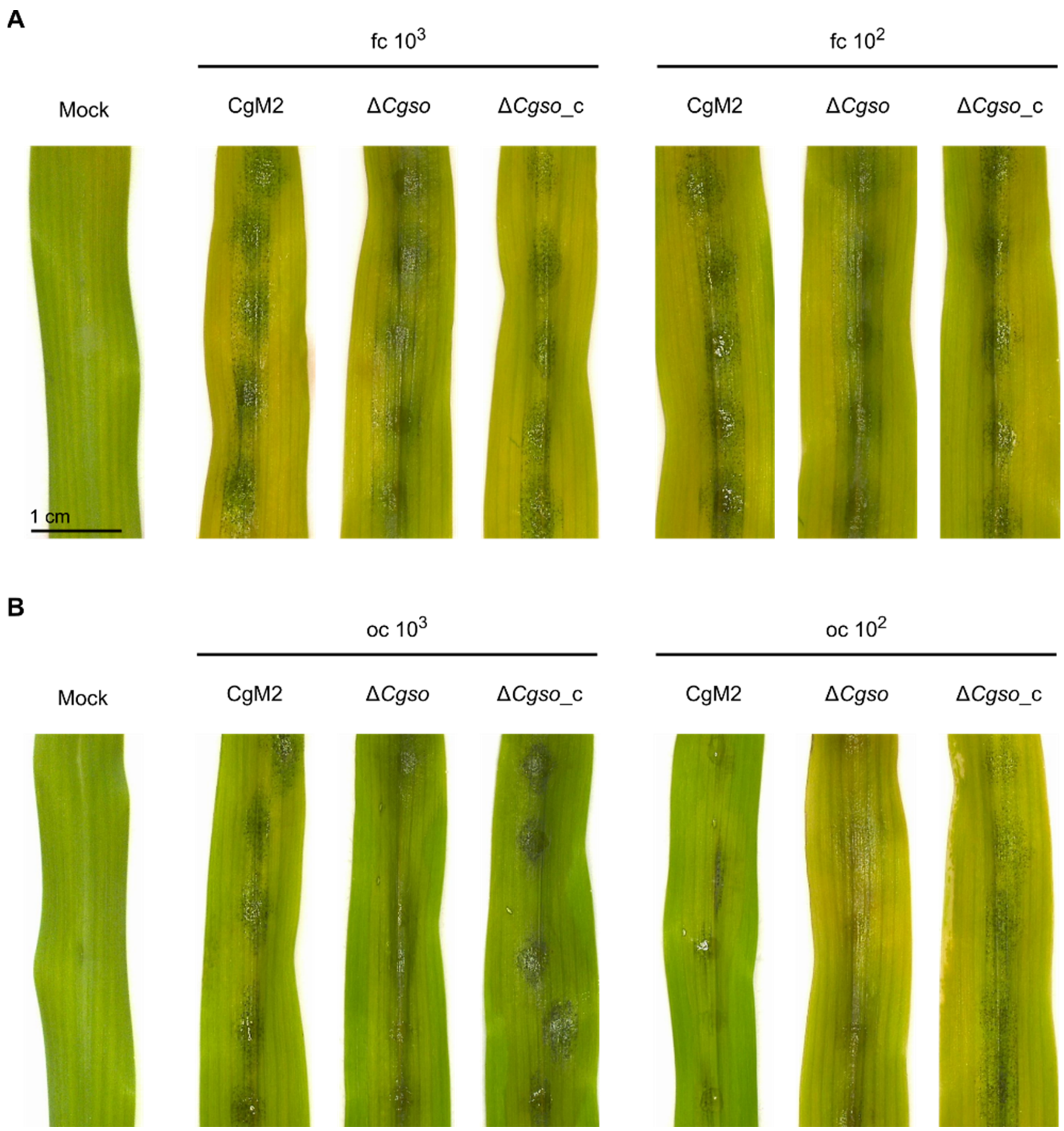

Figure S3. *Zea mays* leaf infection, overview. Second leaves of 16 d old *Z. mays* plants (cv Mikado) were inoculated with five droplets *C. graminicola* conidia containing 10^3 or 10^2 conidia. Typical appearance of symptoms on intact leaves is depicted after incubation with falcate (fc, (**a**)) or oval (oc, (**b**)) conidia for 5 d, scale bar = 1 cm.

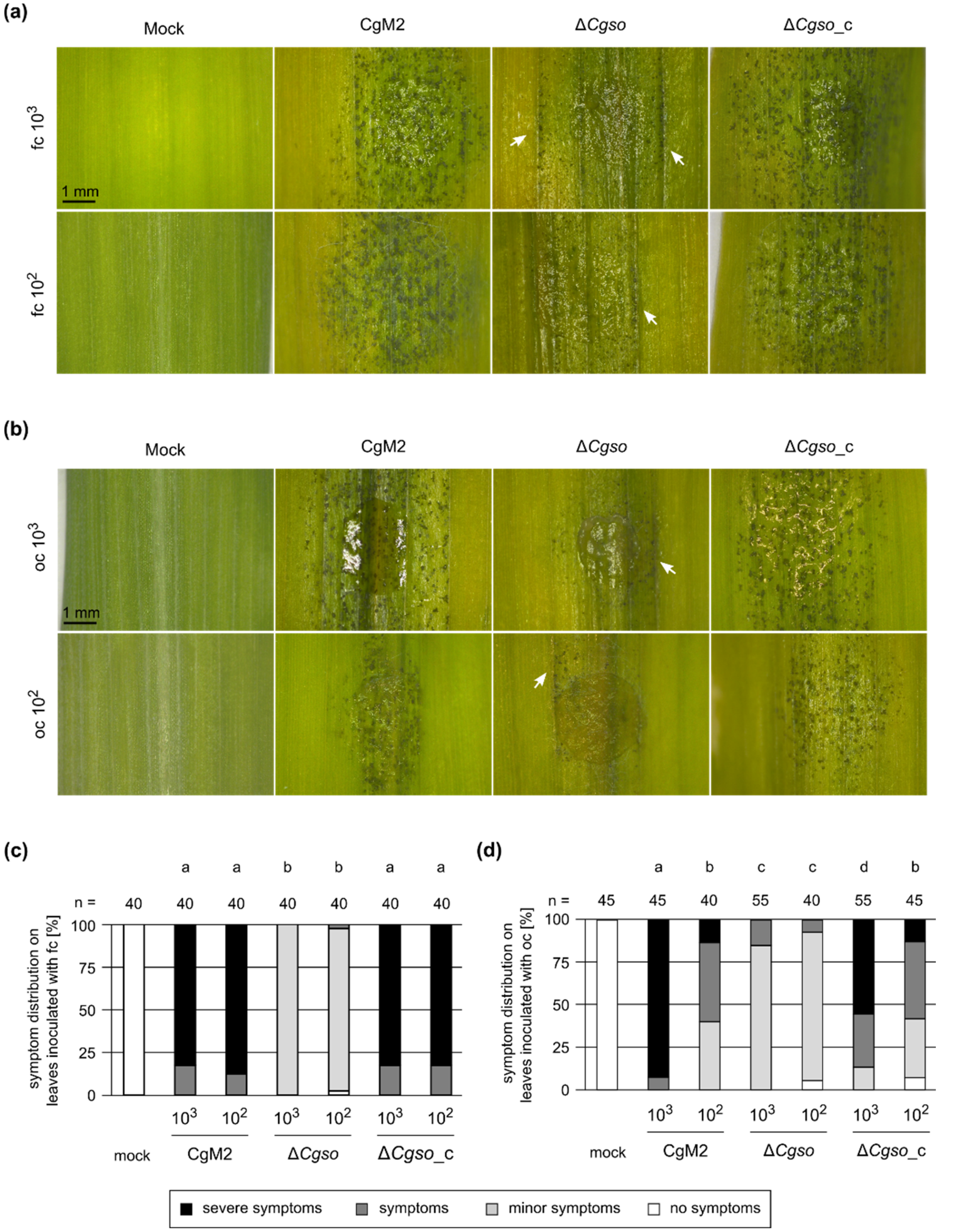

Figure S4. Details and quantification of *Z. mays* leaf infection. Second leaves of 16 d old *Z. mays* plants (cv Mikado) were inoculated with droplets *C. graminicola* conidia containing 10^3 or 10^2 conidia. (**a-b**) Typical appearance of symptoms on intact leaves is depicted after incubation with falcate (fc, (**a**)) or oval (oc, (**b**)) conidia for 5 d. Arrows indicate development of acervuli along vascular bundles, scale bar = 1 mm; (**c-d**) Symptom development caused by falcate (**c**) or oval (**d**) conidia was rated using an established category system [1], n = number of experiments; a, b, c, d, $p < 0.05$

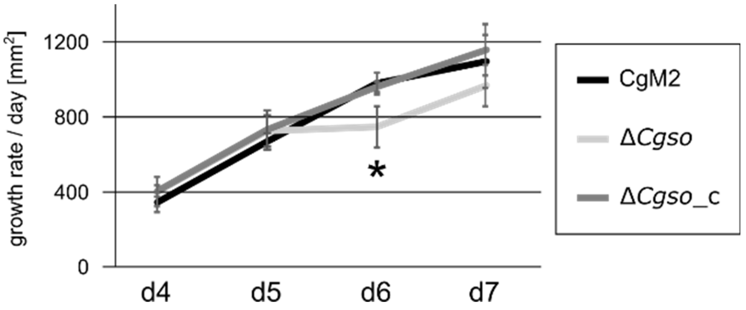

Figure S5. Growth rates of *C. graminicola* strains. *C. graminicola* CgM2 (wildtype), Δ*Cgso* deletion strain as well as complementing strain (Δ*Cgso*_c) were incubated for 7 d on complex medium (CM) plates at 23°C outgoing from a defined inoculum. Starting from day 3, the growth area was optically evaluated using Fiji [2]. Growth rates shown were calculated as the differences of growth areas of two subsequent days. Error bars represent SD calculated from ≥6 experiments, *, $p < 0.05$.

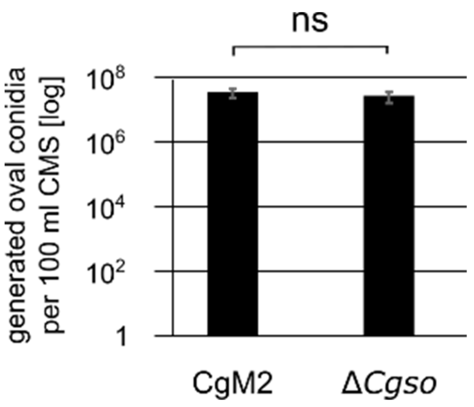

Figure S6. Generation of oval conidia *C. graminicola* wildtype and Δ*Cgso* deletion mutant. Five mycelial plugs of *C. graminicola* CgM2 (wildtype) and Δ*Cgso* were incubated for 7 d in liquid complex medium supplemented with sucrose (CMS) at 23°C in darkness (2 d shaking conditions followed by 5 d incubation without movement). Mycelia and oval conidia were separated by filtering through a sterile cloth. Error bars represent SD calculated from 6 experiments, *, $p < 0.05$, ns = not significant.

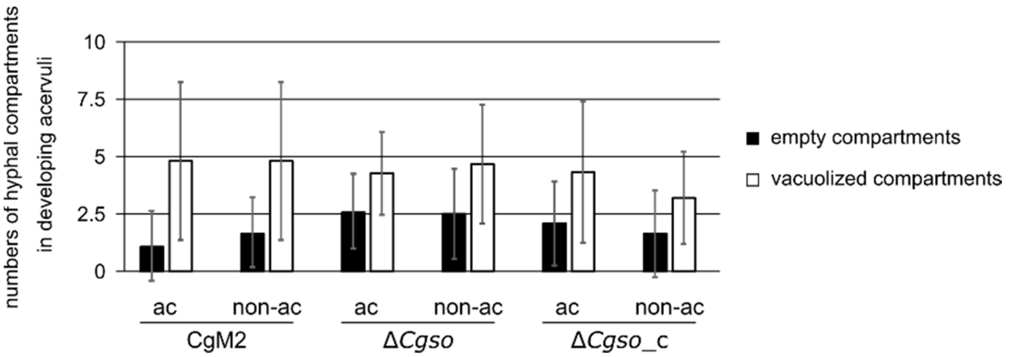

Figure S7. Quantification of empty and vacuolized hyphal compartments in *C. graminicola* hyphae. *C. graminicola* CgM2 (wildtype), Δ*Cgso* deletion strain as well as complementing strain (Δ*Cgso*_c) were incubated for 5 d on reduced oatmeal agar (OMA_{red}) covering microscopic slides at 23°C. Using microscopy, the total number of empty and vacuolized, septae-separated hyphal compartments was estimated for hyphae which show (ac) or do not show (non-ac) developing acervuli. Error bars represent SD calculated from 30 experiments, *, $p < 0.05$.

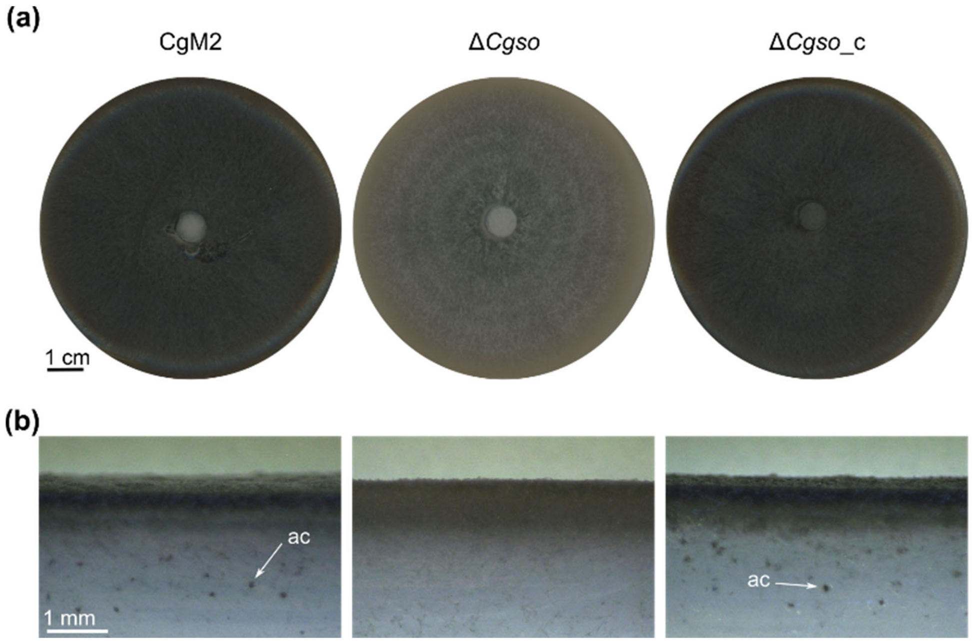

Figure S8. *C. graminicola* falcate conidiation on complex medium. Indicated strains of *C. graminicola* were incubated for 21 d on complex medium (CM) plates at 23°C. (**a**) Plate overview, scale bar = 1 cm; (**b**) cross section, scale bar = 1 mm, ac = acervuli.

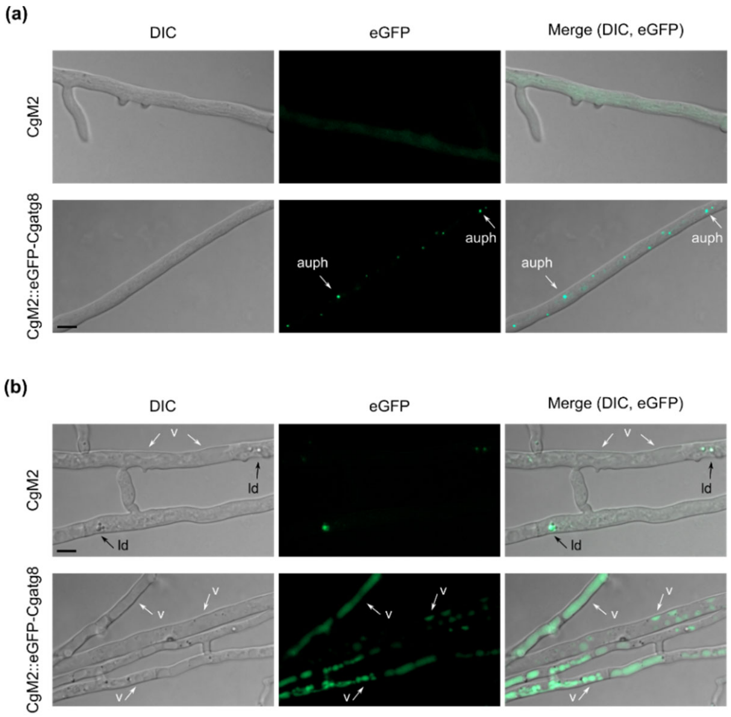

Figure S9. Localization of the autophagy marker protein CgAtg8. (**a-b**) *C. graminicola* wildtype strain CgM2 and CgM2::eGFP-Cgatg8 expressing green fluorescent autophagy marker CgAtg8 were inoculated on microscopic slides covered with reduced oat meal agar (OMA_{red}) for 5 d, 23°C. Localization of eGFP-CgAtg8 (white arrows) in (**a**) young and (**b**) vacuolized hyphae, auph = autophagosome, v = vacuole, ld = lipid droplets, scale bar = 10 µm.

Tables

Table S1. Oligonucleotides used in this study.

Oligonucleotide	Sequence (5′ to 3′)
so_P_fw	**GATCTTCCGGATGGC**CTACTGGATGCCGTCTTTG
so_P_rv	**CGGGAACCAGTTAAC**GTATCTTGCGTTCGACTTGC
so_T_fw	**TCAATATCAGTTAAC**GAAGAAGGGTGAACGACG
so_T_rv	**ATTGTAGGAGATCTT**GTACAAGGAGCTCGTCAACTTC
hph-f	GTTAACTGATATTGAAGGAGCATTTTTGG
hph-r	GTTAACTGGTTCCCGGTCGGCATCTACTC
so_P_comp_fw	**GATCTTCCGGATGGC**GATATCCTACTGGATGCCGTCTTTGC
so_T_comp_rv	**ATGCCCTGCCCCTGA**GATATCGTACAAGGAGCTCGTCAACTTC
nat-1r	TCAGGGGCAGGGCATGCTCA
PtrpC_pJet	**ATTGTAGGAGATCTT**ACTGATATTGAAGGAGCATT
GFP-f	ATGGTGAGCAAGGGCGAGGAGC
GFP-r	CTTGTACAGCTCGTCCATGCCGAGAGTG
Atg8_P_fw	**GTTTTTCAGCAAGAT**GGCAAACTCTGCTAATGAAAAAGGG
Atg8_P_rv	**GCCCTTGCTCACCAT**GTGGACGATGGGAAAGTGTGTT
Atg8_wostart_fw	**GACGAGCTGTACAAG**CGATCCAAGTTCAAGGACGAGC
Atg8_T_rv	**GAGTTCTTCTGAGAT**TTCAGCGCGATGGAACAGATG
so_seq_fw2	CATGTAAGTCGGAAAGCGAGC
so_seq_rv2	GTACCATCAGCTCGTGAGGTT

Bold letters = overhangs, underscored letters = *Eco*RV recognition site

Table S2. Plasmids used in this study.

name of strain	genotype	reference
Colletotrichum graminicola		
CgM2	*C. graminicola* wildtype (wt); also referred to as M1.001	[3]
Δ*Cgso*	Homologous replacement of *Cgso* in CgM2, ssi, hyg^{R}, *Cgso*::*hph*,	this study
Δ*Cgso*_c	Ectopic integration of p*Cgso_c_nat* in Δ*Cgso*, nat^{R}, ssi; Δ*Cgso*::*Cgso*	this study
CgM2::peGFP-Cgatg8	Ectopic integration of peGFP-Cgatg8_gen in CgM2, ssi, gen^{R}, CgM2::*eGFP-Cgatg8*	this study

nat^{R}: nourseothricin resistant, *hyg*^{R}: hygromycin resistant; *gen*^{R}: geneticin-disulfat resistant; *amp*^{R}: ampicillin resistance; *egfp*: gene for enhanced green fluorescent protein (eGFP) of *Aequorea Victoria*; *hph*: hygromycin B phosphotransferase gene; *ura3*: Orotidine-5'-phosphate decarboxylase gene of *S. cerevisiae*

Table S3. *Colletotrichum graminicola* strains used in this study.

name of plasmid	features	reference
pRS-nat	*amp*^{R}, *ura3*, *nat*^{R}	[4]
pRS-hyg	*amp*^{R}, *ura3*, *hyg*^{R}	[5]
pJet1.2	*amp*^{R}	ThermoFisher Scientific
pJet_gen	*amp*^{R}, *gen*^{R}	[6]
pCgso_KO	*5′ Cgso::hph::3′ Cgso, hyg*^{R}, *amp*^{R}	this study
pCgso_c_nat	*5′ Cgso::Cgso::3′ Cgso, nat*^{R}, *amp*^{R}	this study
pJet_nat	*amp*^{R}, *nat*^{R}	this study
peGFP-Cgatg8_gen	*5′ Cgatg8::eGFP::Cgatg8::3′Cgatg8, gen*^{R}, *amp*^{R}	this study

nat^R: resistant to nourseothricin; hyg^R: hygromycin resistant; gen^R: resistant to genetin-disulfat; ssi: single spore isolate; *egfp*: gene for enhanced green fluorescent protein (eGFP) of *Aequorea victoria*;

Table S4. Cellophane penetration ability of *C. graminicola* strains.

	CgM2		Δ*Cgso*		Δ*Cgso*_c	
	n inoculated	n outgrowth	n inoculated	n outgrowth	n inoculated	n outgrowth
replicate 1	3	3	3	3	3	3
replicate 2	3	3	3	3	3	3
replicate 3	3	3	3	3	3	3

References

1. Nordzieke, D.E.; Sanken, A.; Antelo, L.; Raschke, A.; Deising, H.B.; Poggeler, S. Specialized infection strategies of falcate and oval conidia of *Colletotrichum graminicola*. *Fungal Genet Biol* **2019**, *133*, 103276. https://doi.org/10.1016/j.fgb.2019.103276.
2. Schindelin, J.; Arganda-Carreras, I.; Frise, E.; Kaynig, V.; Longair, M.; Pietzsch, T.; Preibisch, S.; Rueden, C.; Saalfeld, S.; Schmid, B.; et al. Fiji: an open-source platform for biological-image analysis *Nat Methods* **2019**, *9*, 676-682, doi:10.1038/nmeth.2019.
3. Forgey, W.M.; Blanco, M.H.; Loegering, W.Q. Differences in pathological capabilities and host specificity of *Colletotrichum graminicola* on *Zea mays*. *Plant Disease Reporter (USA)* **1978**, 573–576.
4. Klix, V.; Nowrousian, M.; Ringelberg, C.; Loros, J.; Dunlap, J.; Pöggeler, S. Functional characterization of MAT1-1-specific mating-type genes in the homothallic ascomycete *Sordaria macrospora* provides new insights into essential and nonessential sexual regulators. *Eukaryotic cell* **2010**, *9*, 894-905.
5. Bloemendal, S.; Bernhards, Y.; Bartho, K.; Dettmann, A.; Voigt, O.; Teichert, I.; Seiler, S.; Wolters, D.A.; Pöggeler, S.; Kück, U. A homologue of the human STRIPAK complex controls sexual development in fungi. *Molecular microbiology* **2012**, *84*, 310-323.
6. Groth, A.; Schunke, C.; Reschka, E.J.; Pöggeler, S.; Nordzieke, D.E. Tracking fungal growth: Establishment of Arp1 as a marker for polarity establishment and active hyphal growth in filamentous ascomycetes. *Journal of Fungi* **2021**, *7*, 580.
